# Pre‑partum blood leukocyte profiles distinguish gestational inflammatory stages that predict birth‑related adverse outcomes

**DOI:** 10.3389/fimmu.2025.1677992

**Published:** 2026-01-07

**Authors:** Hari Shankar, Yash Gupta, Neeta Kumar, William M. Trochim, Almira L. Hoogesteijn, Jeanne M. Fair, Raja Babu Singh Kushwah, Michelle J. Iandiorio, Donthamsetty Nageswara Rao, Ariel L. Rivas

**Affiliations:** 1 Indian Council of Medical Research, New Delhi, India; 2 All India Institute of Medical Research, New Delhi, India; 3 Penn State College of Medicine, Hershey, PA, United States; 4 Cornell University, Ithaca, NY, United States; 5 Cinvestav, Merida, Yucatan, Mexico; 6 Los Alamos National Laboratory, Los Alamos, NM, United States; 7 Texas A&M University, College Station, TX, United States; 8 University of New Mexico, Albuquerque, NM, United States

**Keywords:** adverse birth-related outcomes, immune complexity, inflammatory stages, low birth weight, pattern recognition, preterm birth, prognosis, reductionism

## Abstract

**Introduction:**

Pregnancy is a physiological process accompanied by immuno-dynamic changes (inflammatory stages) that could influence or predict pregnancy outcomes. However, overlapping data intervals among biologically distinct conditions may hinder such differentiation. Here, a retrospective, proof-of-concept study was conducted to (a) differentiate pregnancy-related inflammatory stages, and (b) to prognosticate birth-related double risks (low birth weight and pre-term birth) based on blood tests of pregnant women.

**Method:**

Blood samples collected from 131 Indian pregnant females (192 temporal observations) were retrospectively analyzed with: (1) a reductionist approach, which investigates cell types individually; and (2) a non-reductionist alternative, which uses a proprietary software package to explore pre-partum multicellular interactions and birth-related outcomes. Leukocyte percentages collected during the second and third trimesters were utilized to predict double risks.

**Results:**

While the reductionist analysis failed to distinguish double risks (ambiguity was observed), the non-reductionist method differentiated four inflammatory stages, characterized by: (i) no double risk and a high phagocyte/lymphocyte (P/L) ratio (class ‘A’), (ii) no double risk and a very low P/L ratio (class ‘B’), (iii) 16.6% double risks and a moderately elevated phagocyte/ lymphocyte (P/L) ratio (class ‘C’), and (iv) 83.3% double risks and the highest monocyte percentage (class ‘D’). All double risks observations were associated with statistically higher concentrations of serum ferritin.

**Discussion:**

Combined, longitudinal clinical-inflammatory and personalized data patterns inform whether a pregnancy is associated with double risks and/or when changes occur. Considering pre-partum observations anticipated birth-related outcomes, personalized and prognostic features were demonstrated. Since antenatal care involves routine blood sampling (a low-cost procedure), this methodology is inherently translational. Because construct, internal, external, and statistical validity were supported, if corroborated with prospective studies, this method may assist United Nations’ 2023 goals toward reducing infant mortality.

## Introduction

Pregnancy is a unique physiological process in which maternal immunological responses foster the development of the semi-allogeneic fetus and ensure protection against pathogens (1). Pregnancy-related problems claim the lives of over 295,000 women and more than 223 per 100,000 newborns per year (2). Together with preeclampsia, preterm birth (PTB) and low birth weight (LBW) are the major birth-related adverse outcomes (here described as ‘double risks’) (3).

The magnitude of pregnancy-associated adverse problems is clearly observed in India –where more births occur than in any other country (4). More than half of all births are linked to high-risk factors, such as PTB and LBW (5). While double risks are associated with infant and maternal mortality, there is no precise knowledge on their causation (6). Despite achieving major improvements in the last three decades, India’s infant mortality rates exceeded 25 per 1000 live births in 2016, with the highest values reported in New Delhi and Punjab (7, 8).

The literature on maternal-fetal adverse interactions seems contradictory: while some studies have reported non-significant differences between pre- and post-partum immunological profiles, other reports have claimed that pregnant females express lower percentages of blood lymphocytes (lymphocytopenia) during the second trimester (9, 10). Additional views have suggested that pregnancy begins with a pro-inflammatory stage that, later, transitions into an anti-inflammatory phase and concludes with another pro-inflammatory stage that initiates parturition (11). This seemingly circular and complex progression challenges views that regarded pregnancy as an immunosuppressed process that tolerated allogenic antigens expressed by the fetus (12).

Possible reasons that may explain these contradictory findings include methods of poor validity. While numerous pregnancy-related biomarkers have been explored in blood, they have shown poor sensitivity and/or specificity and have not accurately predicted outcomes associated with late pregnancy (13, 14). While total blood leukocyte counts also have been investigated, count-based tests are prone to distortions induced by extreme (low or high) values. Similarly, percentages are less likely to reveal differences than ratios built from the same datasets (15).

Immunological processes are complex, multidimensional and may display non-normal data distributions, therefore, to detect and distinguish birth-related adverse outcomes, pattern recognition-oriented methods are needed (16, 17). These methods should capture three or more, time-related alternatives, such as no inflammation, early inflammation, late inflammation, and/or the resolution phase of inflammation (18). While several efforts have measured leukocytes (19–22), earlier studies did not explicitly investigate complexity –which characterizes all biological systems and processes (17).

One example of complexity is seen when different temporal scales influence the immune system (23). Another complexity-related feature refers to the differences found between population and personalized analyses. Because individuals differ in clinical history and co-morbidities (and, the environment they have been exposed to), there may be differences between some functions conserved throughout the genetic evolution (i.e., population-specific immunological profiles) and those associated with circadian cycles and/or clinical history which, to be detected, require a personalized analysis (17, 24, 25). Therefore, methodologies expected to capture complexity and dynamics should capture both short-term (rapidly changing) profiles i.e.,#and long-term changes (those expressed as inflammatory stages i.e.,#or phases), i.e., population-level (inflammation-related) and personal (not necessarily inflammation-related) expressions of reproductive health.

New methodological strategies also need to circumvent the problem associated with ‘big data’ approaches, that is, to require large (or very large) ‘sample’ sizes. While such approaches have been explored in reproductive health (26, 27), they are not well fit to investigate dynamic and complex situations characterized by *n* = 1 (personalized medicine) (28).

To improve our understanding on relationships that involve the maternal immune system and birth-related adverse pregnancy outcomes (double risks or PTB and LBW) (29, 30), this study investigated whether prepartum blood leukocyte-related profiles predicted parturition-related adverse outcomes To that end, a pattern recognition-based proprietary software package broadly tested in other biological conditions was utilized (31).

## Materials and methods

### Population and study design

A retrospective longitudinal analysis of pregnancy and birth-related data was performed with blood samples collected from pregnant females attending the antenatal clinics of the Obstetrics and Gynecology departments of either the All India Institute of Medical Sciences (AIIMS), New Delhi, India, or the Post Graduate Institute of Medical Education and Research (PGIMER) of Chandigarh, Punjab, India between year 2009 to 2013. To meet the selection criteria at the time of enrollment, primigravida women had to be (a) aged 19–30 years, (b) without any chronic morbidity, (c) not later than in their second trimester, (d) 8.0–13.0 g/dl of hemoglobin; (e) 18-22 body mass index, (f) middle socioeconomic status (**Table 1**), and (f) willing to participate according to protocol approved by the respective Institutional Ethical Committees. Exclusion criteria included: (a) any metabolic disease, (b) malignancy, (c) heart disease, and (d) any infectious disease. The eligible pregnant women attending their first antenatal visit to the hospital were enrolled using consecutive sampling method until the desired sample size was reached.

**Table 1:** Sociodemographic baseline characteristics of the study participants.

Characteristics	N	Mean ± S.D.
Age	131	23.02 ± 2.55
Height (cm)	131	157.28 ± 5.81
Weight (kg)	131	53.31 ± 8.38
Body Mass Index (kg/m2)	131	21.57 ± 2.86
Systolic Blood Pressure (mm of Hg)	131	110.47 ± 7.99
Diastolic Blood Pressure (mm of Hg)	131	72.26 ± 8.39
Heart Rate (per minute)	131	86.53 ± 9.21
Fasting Glucose (mg/dL)	131	82.94 ± 8.95
Education (N = 131)^*^		
Honors degree	10	7.63
Graduation	59	45.04
Intermediate School	27	20.61
High School	24	18.32
Middle School	9	6.87
Primary School	2	1.53
Occupation (N = 131)^*^		
Professional	11	8.40
Semi-Professional/teacher	11	8.40
Clerical/Shop owner/Farmer	5	3.82
Skilled worker	3	2.29
Semi-skilled worker	4	3.05
Unskilled worker	1	0.76
Housewife	96	73.28

^*^Data expressed in frequency and percentages.

**Figure 1:**
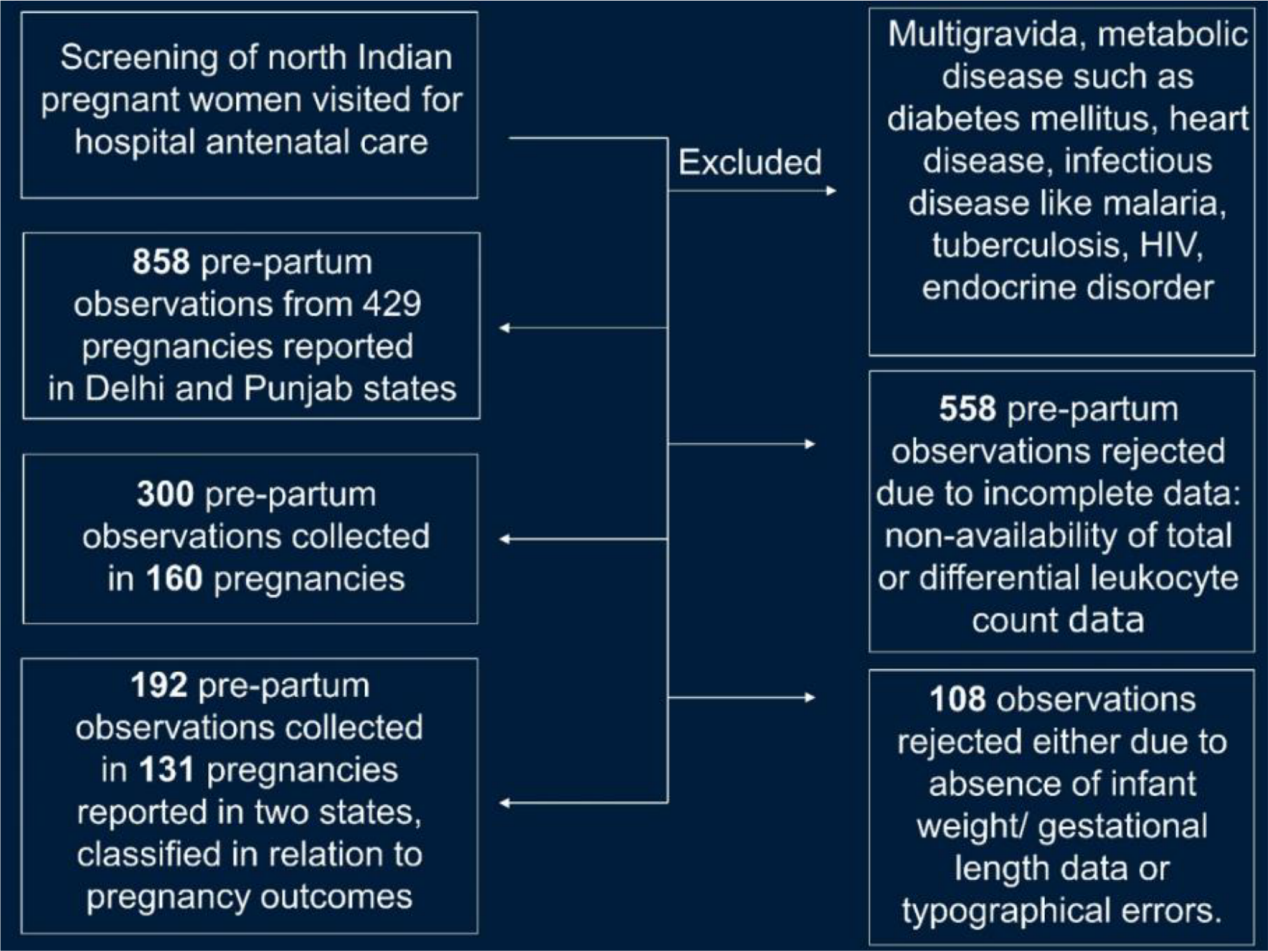
Inclusion and exclusion criteria of the study group. A retrospective dataset was meant to facilitate a preliminary evaluation of an immunologically grounded, prognostic (prepartum based), diverse (two-state), highly translational, and personalized method on maternal-fetal relationships.

Out of 429 candidate pregnancies (which included 858 observations), 558 observations were rejected due to incomplete differential leukocyte data. Additionally, these samples had inconsistencies such as sample collection time outside the enrollment gestation window or lack of documentation about sampling timing. Some samples had unrealistic values in the dataset, might be due to typographical errors, and a few laboratory reports falsely altered leukocyte counts probably due to sample hemolysis. The remaining 300 observations were gathered from 160 pregnancies that contributed up to two temporal observations. However, 108 of such observations lacked information on neonates. Thus, the prognostic evaluation was conducted with 131 pregnancies, which generated 192 prepartum observations that retrospectively determined whether immunological (prepartum) maternal observations predicted birth-related adverse outcomes (**Figure 1**). Low birth weight (< 2500 g) and preterm birth (gestation length < 259 days) were described as “double risks”. **Supplementary Data Table 1** reports the 192 prepartum observations of this retrospective study, indicating the blood total leukocyte count (TLC/mm^3^) and leukocyte percentages of each observation as well as serum ferritin and C-reactive protein (CRP) concentrations (**Figure 1**).

### Methodological procedures

Two methods were compared: (1) a reductionist one, which assessed isolated variables; and (2) a non-reductionist alternative, which explored interactions among variables (15, 16, 31).

### Laboratory procedures

As described elsewhere (32), 5 ml blood samples were collected with an automated cell counter (Sysmex XN1000, Japan). Aliquots were taken to conduct (i) complete blood cell counts and differentials, and (ii) serological studies (including the concentrations of ferritin and CRP).

### Validation

To explore the construct validity of the non-reductionist method (33), immunology-based data partitioning was compared to independent biological variables (ferritin and CRP) –an exploration that also considered statistical validity. To explore the internal and external validity (repeatability across metrics and across populations), blood samples collected from individuals residing in two (New Delhi and Punjab) territories of India were investigated with several data structures.

### Detection and evaluation of complex immunological data patterns

Complex data structures were created and analyzed as described elsewhere (34, 35). Interactions among blood leukocyte were explored with indicators created by a proprietary algorithm (US patent 10,429,389 B2), which revealed patterns that facilitated data partitioning into three or more data groups that (a) partially or totally revealed non-overlapping intervals, and (b) displayed qualitatively different biological conditions, such as PTB and LBW, i.e., double risks.

### Sample-size estimation

Because immunological responses are known to be non-linear, no previous study on the dynamic and complex immunological interactions associated with pregnancy was available to generate estimates. Furthermore, because personalized assessments were pursued (where *n* = 1), this study could not estimate the sample size required to evaluate the novel method. However, previous studies on several (non-pregnancy-related) conditions have shown that, when the internal complexity and dynamics of immunological responses are evaluated, they can reveal three or more data groups that display non-overlapping data intervals (and, consequently, achieve statistically significant differences) even when the sample size is as low as *n* = 6 (15). Because abundant, biologically interpretable, longitudinal data patterns have been observed when *n* < 101 (16, 34, 35), it was expected that a sample with *n*>130 was likely to show distinct data patterns, if they existed.

### Statistics

Comparisons among proportions were conducted with the chi-square test and medians were compared with the Mann-Whitney test. Such analyses were performed with a commercial package (Minitab LLC, 2024). P-values less than 0.05 were considered statistically significant.

### Ethics statement

In compliance with the 1964 Helsinki Declaration and later amendments, this study was approved by the Ethics Committees of the two participating institutions (AIIMS, approval no. IEC/NP-339/2010; and PGIMER, approval No. 10/4815).

## Results

### Reductionist analysis

**Figure 2:**
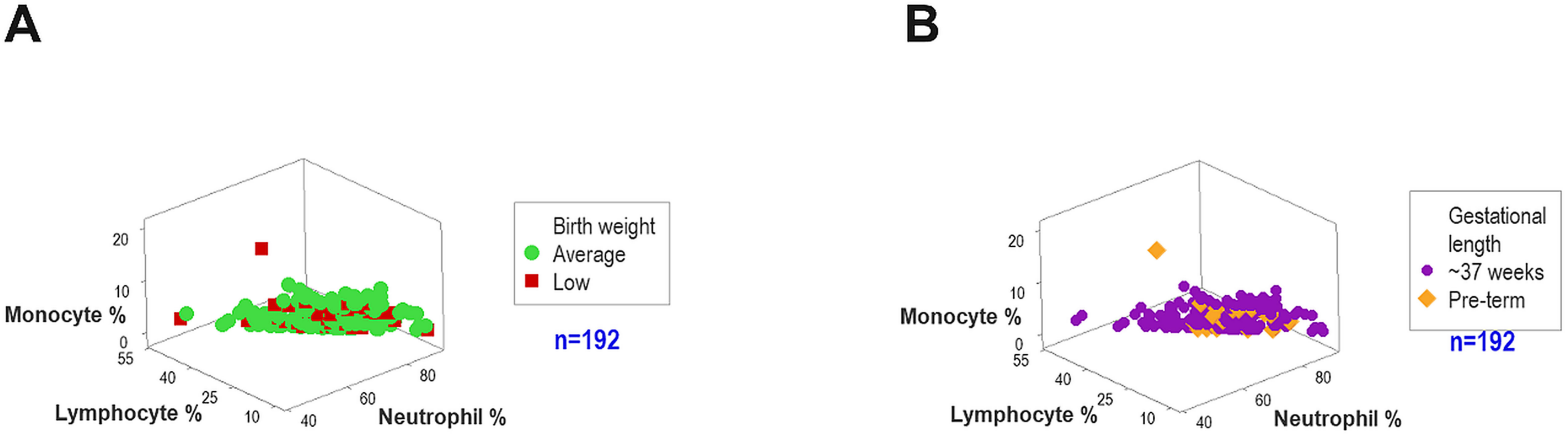
Reductionist analysis. The analysis of separate cell types was associated with overlapping, non-discriminant data intervals. Neither birth weight **(A)** nor gestational length **(B)** was differentiated by leukocyte cell types.

The reductionist approach focused on separate variables (e.g., cell types). Overlapping data intervals of lymphocyte, monocyte, or neutrophil percentages prevented discrimination of subsets associated with birth weight (**Figure 2A**) and gestational length (**Figure 2B**).

### Non-reductionist analysis –preliminary findings and validation

**Figure 3:**
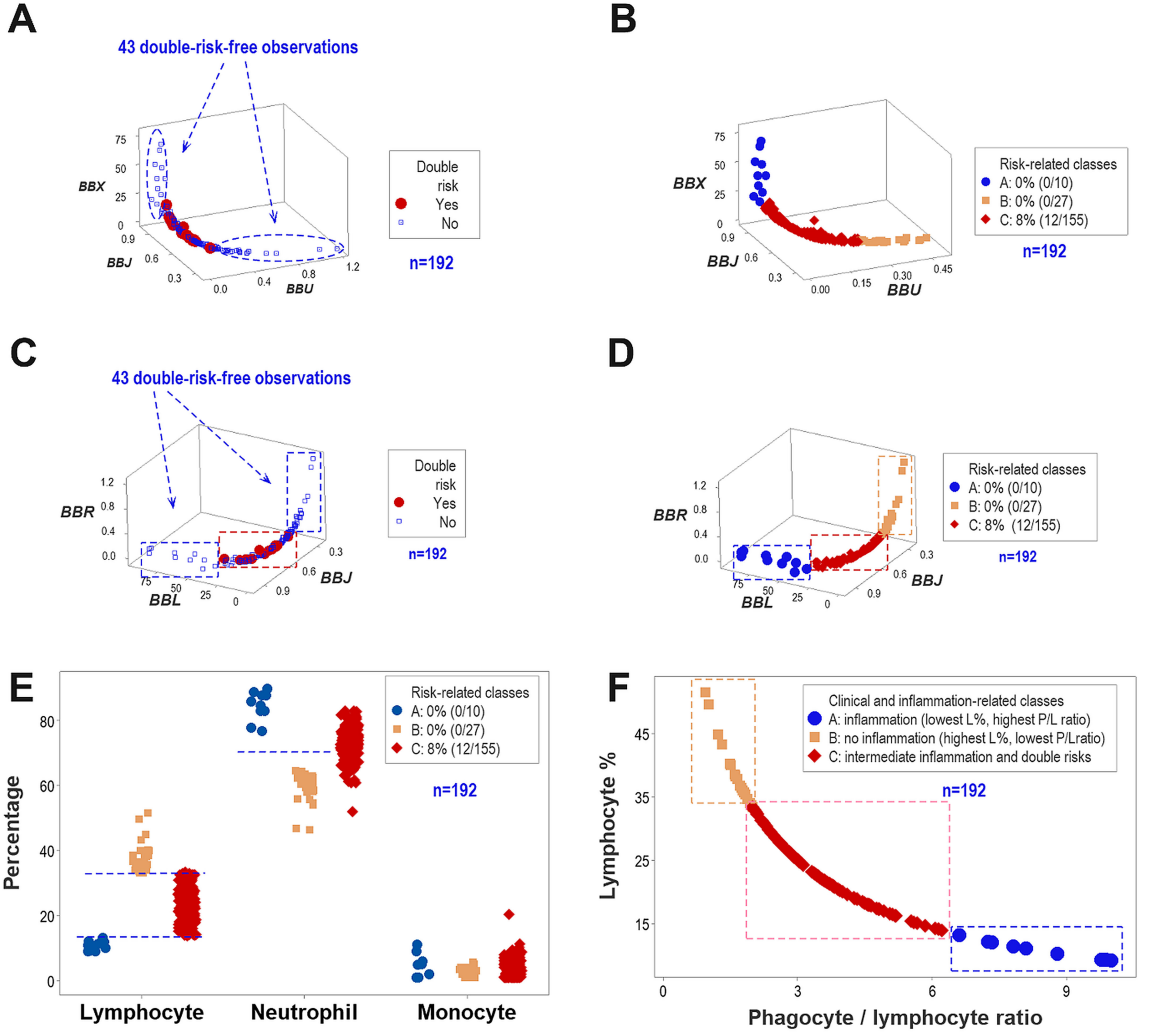
The non-reductionist method. The analysis of complex data interactions among leukocytes revealed a non-random distribution of most double risks (PTB, LBW) and two groups only composed of double-risk-free observations **(A)**. Consequently, the data were partitioned into three non-overlapping data groups, with double risks representing 7.7% of all group C observations **(B)**. Because a partially different data structure revealed similar findings, internal validity was supported **(C, D)**. Data partitioning was biologically validated with additional analyses, which showed that both the lymphocyte percentage (L%) **(E)** and the relationship between the phagocyte/lymphocyte (P/L) ratio and the L% **(F)** distinguished all three groups of observations.

The three-dimensional analysis of complex indicators displayed distinct spatial features (such as orthogonal data inflections) that offered interpretable information; e.g., double-risk-free data points were clustered (**Figure 3A**). Such patterns helped partition the data into three non-overlapping groups, of which only one (group ‘C’) reported all double-risk data points (**Figure 3B**).

Findings did not depend on any one data structure: the same inferences were supported by several data structures that displayed distinct patterns (**Figures 3C, D**). The data partitioning process was validated twice: a unidimensional analysis showed that the lymphocyte (L) percentage distinguished all three data groups (**Figure 3E**) and a bi-dimensional analysis demonstrated that, together, a ratio and a percentage also generate non-overlapping intervals (**Figure 3F**).

### Non-reductionist, population-based (*clinico-inflammatory*) stages

**Figure 4:**
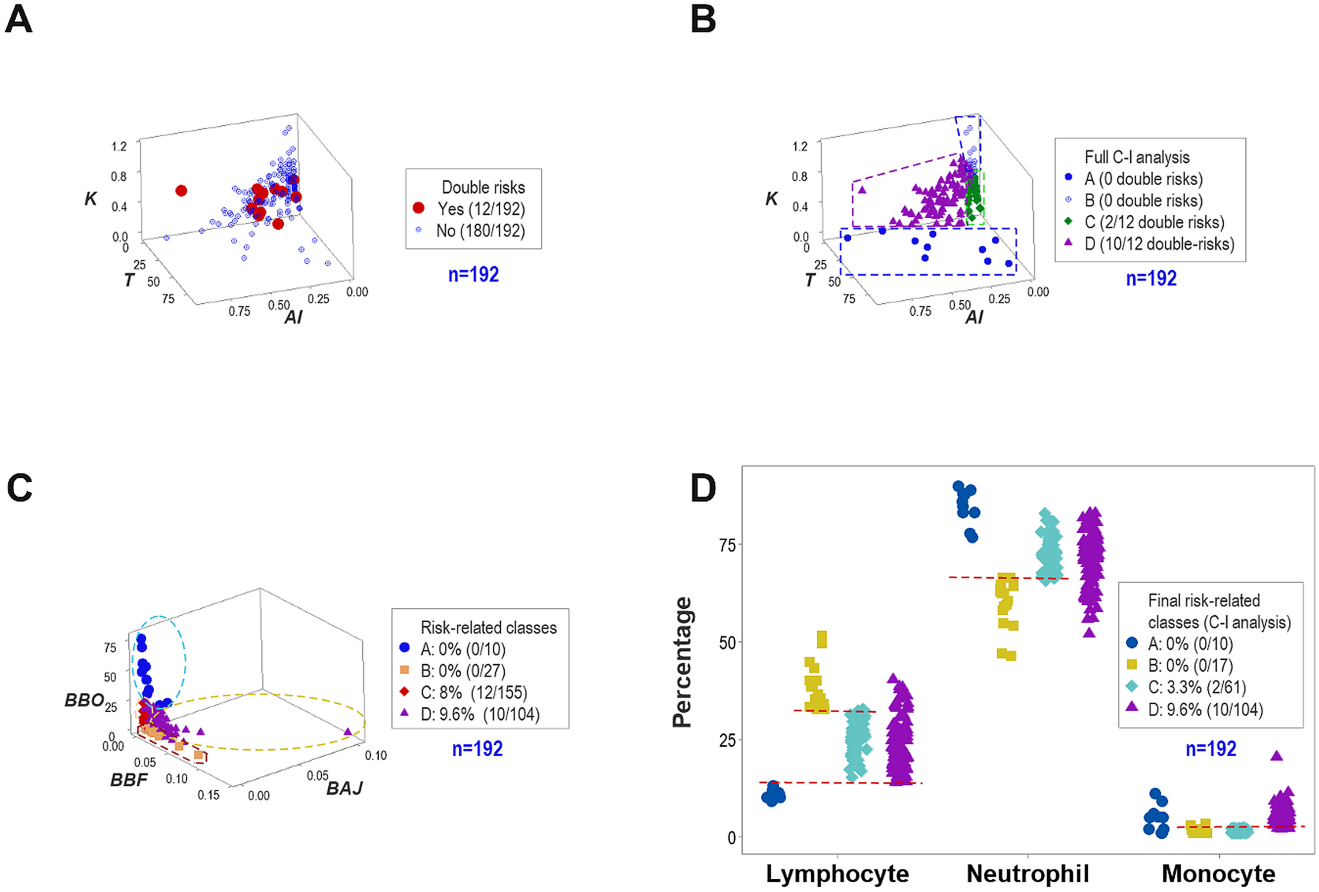
The non-reductionist, population-based (*clinico-inflammatory*) analysis. Additional analyses revealed four non-overlapping data groups, which revealed patterns consistent with four **(A-D)** inflammatory stages **(A)**. Two of the 12 double-risk (16.6% of all double-risk) observations were classified within the ‘C” group and 10/12 of such data points (83.3%) were found within the ‘D’ group **(B)**. Data partitioning also revealed internal validity: a different data structure supported the same data classes **(C)**. The biological validation of the four-class classification indicated that the L% differentiated classes A-C while the monocyte percentage (M%)and L% distinguished class D from all other data groups **(D)**. Because class D displayed **(a)** higher M% than classes B and C and **(b)** higher L% than class A, class D observations seemed to express the resolution phase of the inflammatory process. Because class D reported 83.3% (10/12) of all double risks and this group represented a non-trivial percentage (9.6%) of all observations, it was concluded that the 4-class partitioning process was the most informative for these data.

Four non-overlapping data groups were distinguished when several data structures were assessed, and the differentiation of inflammatory stages was emphasized (**Figures 4A, B**). Such analysis divided former group ‘C’ into two subsets (now named ‘C’ and ‘D’, **Figure 4B**). The new group ‘C’ included two of the 12 double-risk observations (16.6% of all double risks) while group ‘D’ captured all other double risks (10/12 or 83.3% of all double risks, **Figure 4B**). This population-based, *clinico-inflammatory* analysis was validated by a separate data structure (**Figure 4C**).

The biological validation corroborated earlier findings in reference to groups A-C (all distinguished by the L%) and justified the creation of group ‘D’ (which showed higher monocyte percentages than groups ‘B’ and ‘D’, and higher L% than group ‘A’, **Figure 4D**).

### Non-reductionist, personalized (and temporal) stages

**Figure 5:**
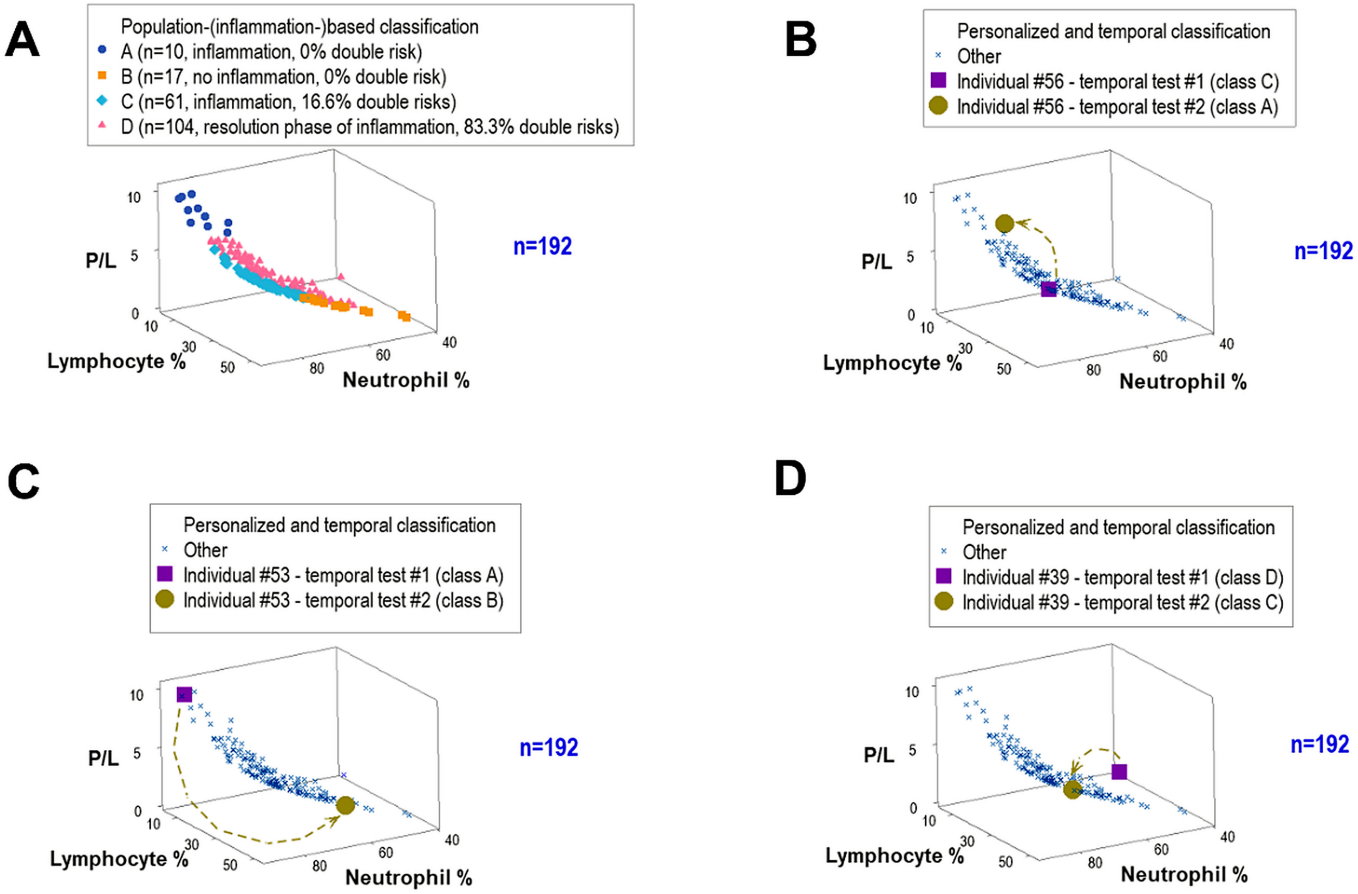
The non-reductionist, personalized analysis. The inflammatory stage-based, population-grounded approach **(A)** was compared to personalized assessments **(B-D)**. While three-dimensional analysis of biologically interpretable variables investigated under the population-based version did not distinguish all data groups **(A)**, similar three-dimensional analyses that only considered temporal data from one individual exhibited trajectories or directionalities that connected all groups characterized by inflammatory stages. For example, consecutive observations connected: class “C” with class “A” data points (individual #56, **B**); class ‘A’ with class ‘B’ observations (individual #53, **C**); and class ‘D’ with class ‘C’ observations (individual #39, D). Findings are consistent with the hypothesis that feedback-like (circadian) cycles express phenomena at temporal scales too small, highly dynamic (changing) and/or too heterogeneous to be detected by inflammatory stage-based analytics. Supporting this hypothesis, the median blood prepartum ferritin levels were significantly higher in pregnancies that resulted in partum-related ‘double risks’ than in those that did not experience ‘double risks.’

Two variations of the non-reductionist model were compared. The one based on population data (which reflected inflammatory stages, **Figure 5A**) was compared to a personalized-temporal alternative (**Figures 5B–D**). Such analyses revealed that a very high proportion of prepartum observations (between 90.4 and 100%) did not evolve into double risks. The analysis of *temporal data directionality* revealed connections between: (a) C with A, (b) A with B, and (c) D with C class data points (**Figures 5B–D**). While the four clinical-inflammatory data classes did not offer temporal information, the version that measured personalized data directionality distinguished pregnancy-specific likely outcomes even when the earlier tests pointed at a different prognosis.

Three examples documented the previous statement. As shown in **Figure 5 B**, an earlier assessment (originally classified within the class ‘C’ group, which included 16.6% of all double risks) was later grouped within the class ‘A’ group (which was associated with 0% double risks).

Similarly, a pregnancy initially classified within the class ‘A’ group was later included in the class ‘B’ group (also associated with 0% double risks, **Figure 5C**). Consecutive observations also connected class ‘D’ with class ‘C’ data points (which included double risks, **Figure 5D**).

The assessment of a major co-morbidity was non-informative. In the population under study, both the reductionist and the non-reductionist approaches failed to distinguish pregnancies when anemia-related classes were investigated (**Supplementary Figures 1 A, B**).

Statistical validity was supported. A Mann-Whitney test that compared the median monocyte percentages of data groups C and D revealed non-overlapping data intervals and values approximately 5 times higher in the D than in group C observations (*p* < 0.01, **Supplementary Figure 2**).

External validity was also explored. There was no significant difference in the proportion of New Delhi and Punjab double-risk cases (*p*>0.5, chi-square test, **Supplementary Figures 3A, B**).

Additional findings supported the construct and statistical validity of the non-reductionist method and tested two expressions of inflammation. Immunological findings were corroborated by serum ferritin concentrations of pregnant females, which reached statistically significant higher values in double risks than in non-double risk observations (36.65 and 20.29 and μg/L, respectively; *p<*0.02, Mann-Whitney test, **Supplementary Figure 4A**). While ferritin (in other biological processes) is a well-known inflammation marker, the concentration of a classic marker of inflammation (CRP) did not differ between double-risk-positives and -negatives (**Supplementary Figure 4B**).

## Discussion

This is the first report of an operational method that explores dynamic and complex immunology in maternal-fetal interactions. Findings supported the view that prepartum immunological profiles may predict LBW and PTB. Such information may facilitate early and personalized medical responses. System‑level (e.g., population‑level) information does not necessarily reflect personalized conditions (which are influenced, at least, by personal comorbidities, clinical history and/or environments where each individual lives or has lived). It is suggested that both (population-level and personalized) analyses are needed (17).

### Biomedical perspectives

As indicated in **Figure 4D**, findings supported the view that a relatively high proportion of monocytes may be associated with premature birth (6). This finding also suggests that early occurrence of the inflammation resolution stage may be triggered by double risks.

While earlier methodologies have been mainly binary (i.e., reductionist) and, therefore, only pro- and anti-inflammatory perspectives have been considered (6), the non-binary method here explored suggests that at least four inflammatory stages may occur, including the resolution phase of inflammation. Such a finding appears to differ from earlier studies that proposed three inflammatory stages with a binary, unidirectional process composed of pro-inflammatory→ non-inflammatory→ pro-inflammatory immuno-temporal phases (17, 36).

The multiple temporal trajectories exhibited by the personalized non-reductionist version is consistent with circadian-like processes (37–39). Hence, immune responses may occur in a circular, not in a linear fashion (17). When bio-temporal stages are not considered, assessing any variable just once may promote confounding because the same value of the same cell type may be found in different inflammatory stages. This potential error is further aggravated when the same cell type participates in different (even opposite) immunological functions, e.g., monocytes both promote and destroy neutrophils within 48 hours (40). Therefore, to prevent misclassifications, tests that both identify inflammatory stages and detect personalized processes over time are needed.

### Methodological perspectives

The informative advantages of combinatorial methods were documented. Highlighting interactions instead of isolated entities allowed the method to capture internal complexity –a feature that facilitates statistical testing even with small sample sizes (41).

Validation was based on biologically interpretable information. Convergent, construct, internal, external and statistical validity were empirically supported (33, 42).

Construct validity (i.e., to measure what is biologically relevant, not just what is conveniently measured) was supported by the correlation observed between a separate and independent variable (i.e., serum ferritin concentrations) and double-risk observations. In the last three decades, high ferritin and double-risk observations have been reported in several countries (43–45). However, earlier studies have not simultaneously assessed ferritin with CRP concentrations.

In severe COVID-19 cases, high ferritin serum levels have also been associated with hyper-inflammation (46). Together with ferritin levels, leucocyte profiling have been claimed to differentiate inflammatory disorders and conditions linked to iron overload (47). Yet, high ferritin concentrations do not always predict inflammation: high ferritin-associated double risks may occur even when no inflammation (low C-reactive protein values) are observed (44). Thus, reductionist inferences (of the type ‘high ferritin always indicates inflammation’) were not supported. Instead, these findings support a revision of the *meaning of inflammation* in reproductive health.

Convergent and construct validity were documented by two data structures that, once analyzed by the same algorithm, partitioned the data into the same subgroups (**Figures 3A–D**) (42). Such operationalization also indicated construct validity: the concept being measured (immunological dynamic complexity) informed about clinically relevant outcomes, such as LBW and PTB.

The elimination of data overlapping demonstrated internal validity confounding was prevented even when different data structures were used (48). Because pregnancies that took place in two Indian states (New Delhi and Punjab) did not reveal obvious birth-related differences, external validity was also supported (**Supplementary Figure 3**).

The non-overlapping intervals displayed by the four inflammatory stages revealed statistical validity (**Figure 3D**). Statistical validity was also shown by the differences found between high-ferritin, double-risk-associated and low-ferritin, non-double-risk-associated observations (*p* < 0.02, Mann-Whitney test, **Supplementary Figure 4**).

The non-reductionist methodology has already been validated in many other (non-reproduction-related) contexts (15, 16, 24, 25, 31, 49). Given such robustness, the potential applicability of this method in reproductive health may be substantial. If further corroborated, this approach may support United Nations’ 2030 Sustainable Development Goals toward reducing infant mortality (50).

### Caveats

This study did not consider the potential different speed that blood cells may display in blood vessels. Such an omission may matter because (a) neutrophils move much faster than mononuclear cells (19.4 vs. 9.6 μm/min, respectively), and (b) lymphocytes move twice faster than monocytes (which circulate at, approximately, 4.2 μm/min) (51, 52). Consequently, data collected with blood tests (at a given site, at a given time point) do not necessarily reflect the spatial-temporal differences likely to occur among leukocyte-leukocyte interactions at inflammatory sites (53). Another potential limitation is the exclusion of samples that did not meet minimum quality requirements necessary for valid leukocyte-based prognostic modelling; however, all exclusions were based entirely on data integrity issues, and not based on participant characteristics.

Furthermore, this study only investigated blood leukocytes. Because blood only contains 2% of all leukocytes, findings do not necessarily represent the whole immune system (54, 55).

## Conclusions

Birth-related adverse outcomes were associated with late inflammations. Because pregnancy-related data on blood leukocytes are abundantly available, combinatorial (non-reductionist) methods can be rapidly corroborated and potentially applied without new technologies or training programs.

## Data Availability

The original contributions presented in the study are included in the article/**Supplementary Material**. Further inquiries can be directed to the corresponding authors.

## Supplementary material

Supplementary Figure 1 Co-morbidities–Assessment of anemia. Anemia was not suspected to explain the observed findings: similar percentages of anemic pregnancies were detected by the classic (percentage-based, **A**) and the non-reductionist (complex indicated-based) approach **(B).**

Supplementary Figure 2 Demonstration of statistical validity. A Mann-Whitney test compared the median monocyte percentages of data groups C and D. Non-overlapping data intervals revealed that group D values were approximately 5 times higher than those of group C (*p* < 0.01). This indicates that the final addition to the clinical-inflammatory analysis (group D, a subset of former group C) captures the resolution phase of the inflammatory process, i.e., when the monocyte percentage reaches its highest values.

Supplementary Figure 3 Construct and external validity. The construct validity of the non-reductionist approach was supported: the proportion of double risks did not differ among subpopulations –it was 60% (17/28) and 40% (11/28) in New Delhi and Punjab females, respectively (*p*>0.05, Chi-square test, **A**). External validity was also documented: similar findings were observed in two separate states **(B).**

Supplementary Figure 4 Validation with independent (non-leukocyte) variables and assessment of inflammation. Immunological profiles were corroborated by ferritin concentrations, which showed a statistically higher median in double-risk than in non-double-risk observations (*p* < 0.02, Mann-Whitney test, **A**). In contrast, CRP did not differ between double-risk positives and negatives **(B).**
