# Supplementary figures and images for: Pre‑partum blood leukocyte profiles distinguish gestational inflammatory stages that predict birth‑related adverse outcomes
Source: Front Immunol. 2026 Jan 7;16:1677992. doi: 10.3389/fimmu.2025.1677992 (PMC12819648; doi:10.3389/fimmu.2025.1677992)

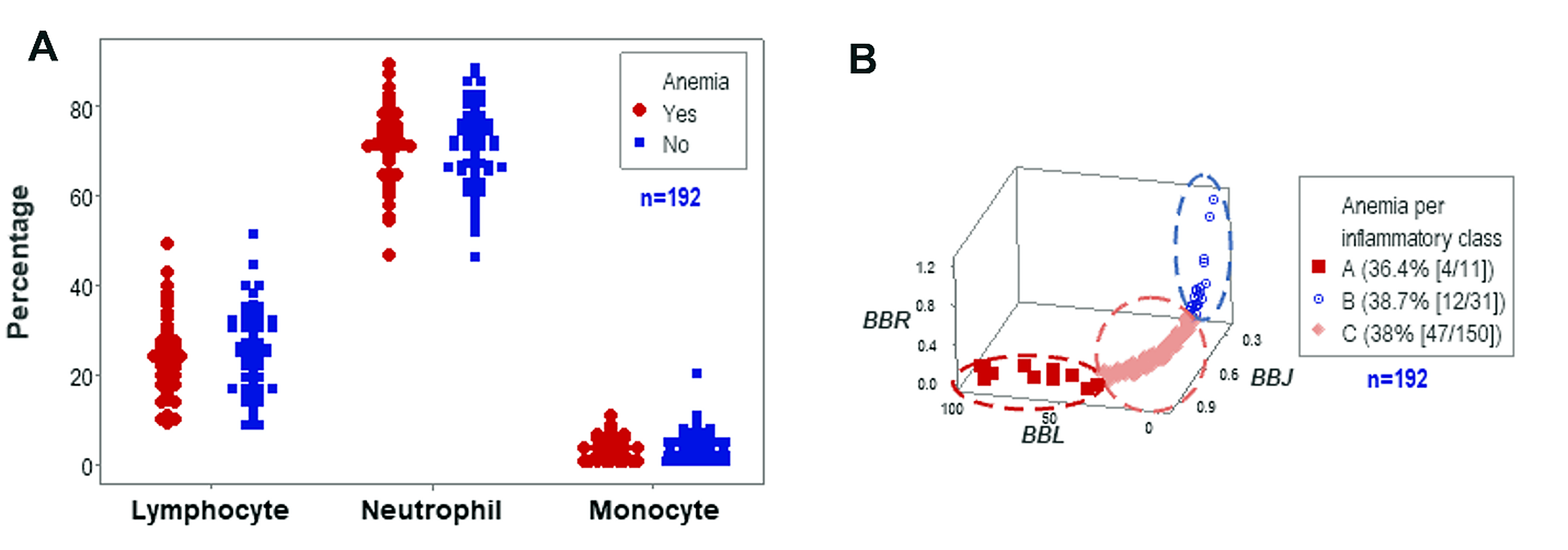

Supplement: Supplementary Figure 1 — Co-morbidities–Assessment of anemia. Anemia was not suspected to explain the observed findings: similar percentages of anemic pregnancies were detected by the classic (percentage-based, A) and the non-reductionist (complex indicated-based) approach (B). [file Image1.png]

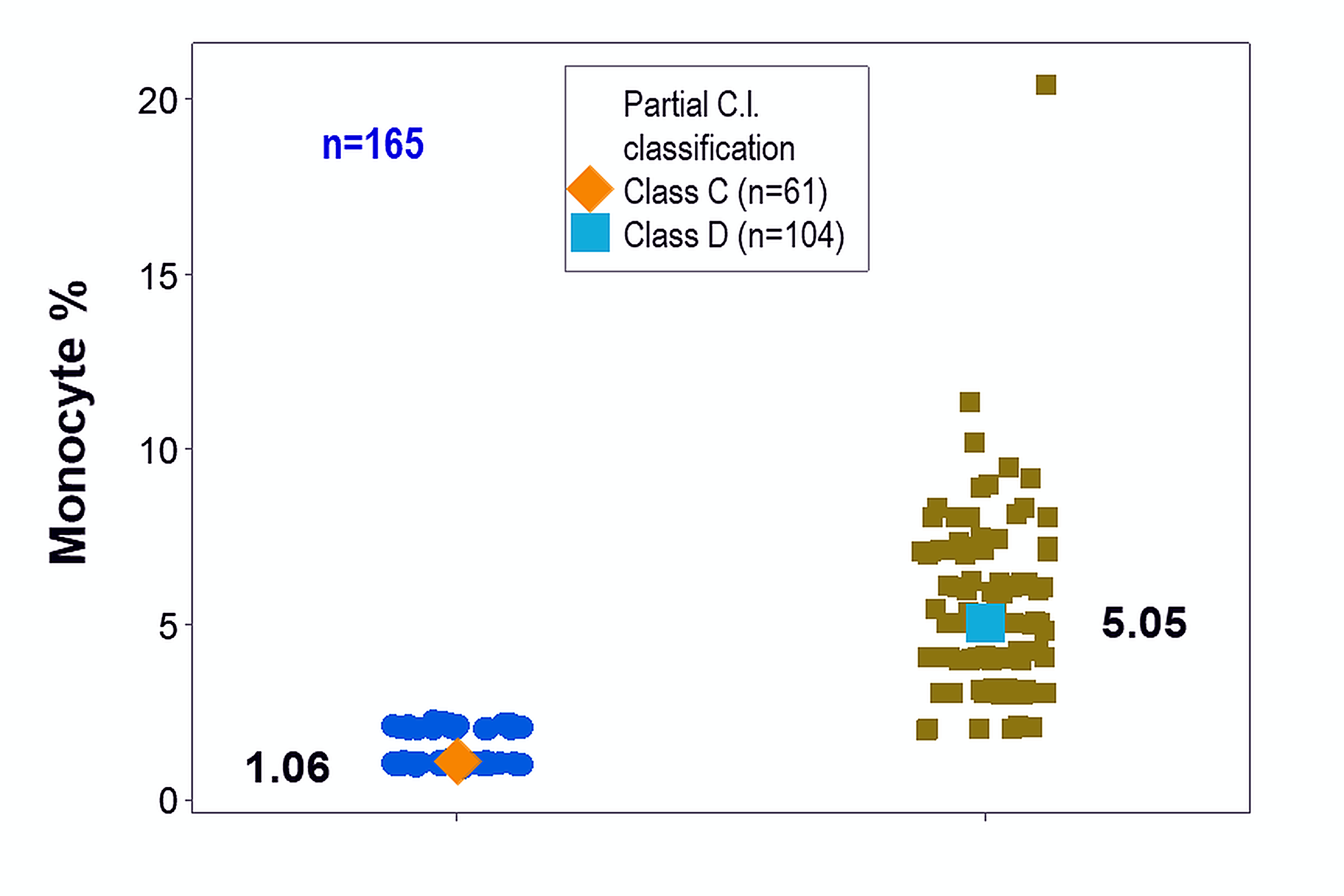

Supplement: Supplementary Figure 2 — Demonstration of statistical validity. A Mann-Whitney test compared the median monocyte percentages of data groups C and D. Non-overlapping data intervals revealed that group D values were approximately 5 times higher than those of group C (p < 0.01). This indicates that the final addition to the clinical-inflammatory analysis (group D, a subset of former group C) captures the resolution phase of the inflammatory process, i.e., when the monocyte percentage reaches its highest values. [file Image2.png]

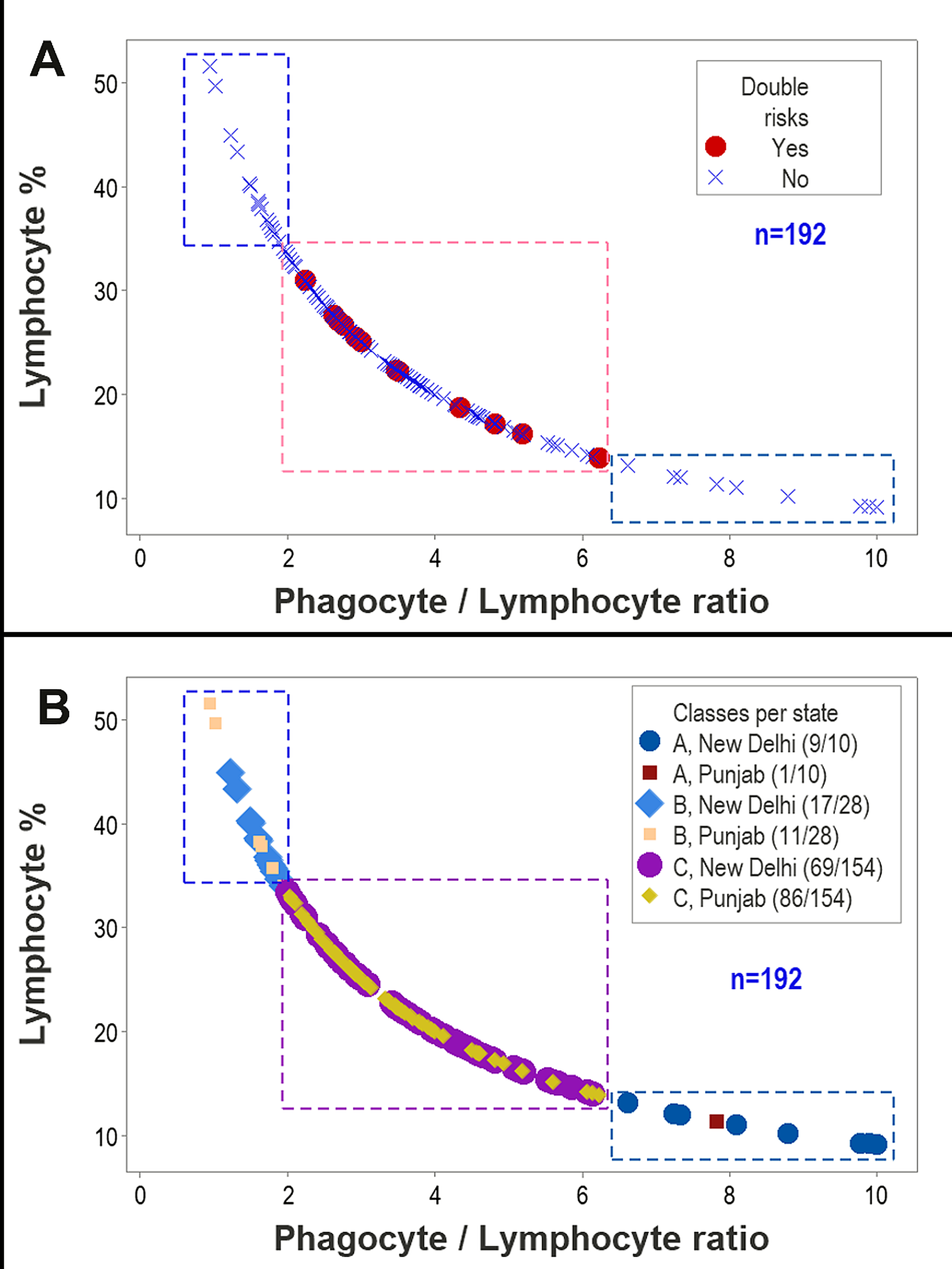

Supplement: Supplementary Figure 3 — Construct and external validity. The construct validity of the non-reductionist approach was supported: the proportion of double risks did not differ among subpopulations –it was 60% (17/28) and 40% (11/28) in New Delhi and Punjab females, respectively (p>0.05, Chi-square test, A). External validity was also documented: similar findings were observed in two separate states (B). [file Image3.png]

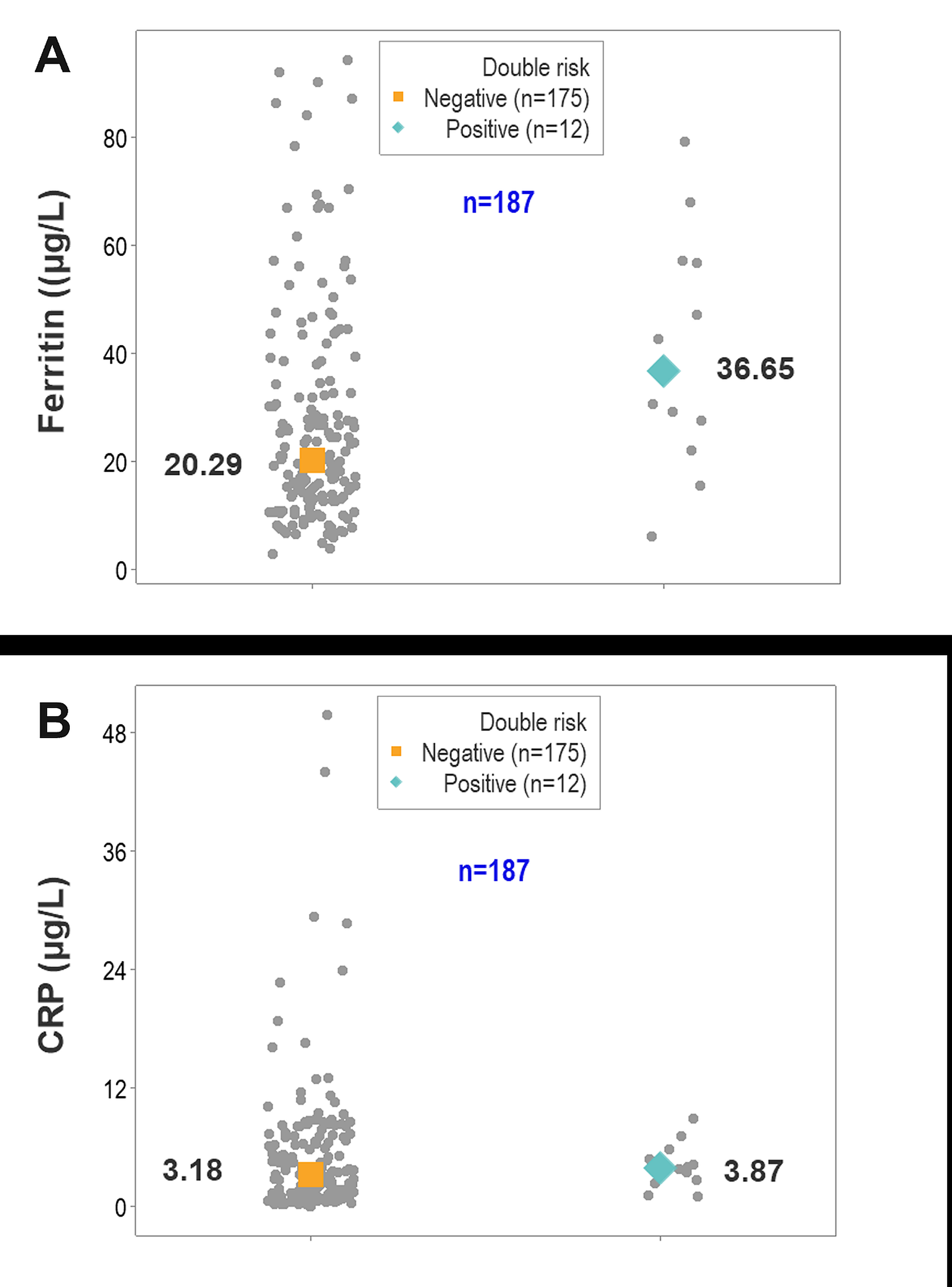

Supplement: Supplementary Figure 4 — Validation with independent (non-leukocyte) variables and assessment of inflammation. Immunological profiles were corroborated by ferritin concentrations, which showed a statistically higher median in double-risk than in non-double-risk observations (p < 0.02, Mann-Whitney test, A). In contrast, CRP did not differ between double-risk positives and negatives (B). [file Image4.png]
